# Influence of electrolyte imbalance on regional wall motion abnormalities in STEMI patients of North Indian origin

**DOI:** 10.3389/fcvm.2023.1223954

**Published:** 2023-11-30

**Authors:** S. Mohd. Shiraz Rizvi, Sini Sunny, Irshad A. Wani, Farzana Mahdi, Zeeshan H. Zaidi, Namakkal S. Rajasekaran

**Affiliations:** ^1^ Department of Biochemistry, Era’s Lucknow Medical College & Hospital, Era University, Lucknow, India; ^2^ Cardiac Aging & Redox Signaling Laboratory, Molecular and Cellular Pathology, Department of Pathology, Birmingham, AL, United States; ^3^ Department of Cardiology, Era’s Lucknow Medical College & Hospital, Era University, Lucknow, India; ^4^ Department of Community Medicine, Era’s Lucknow Medical College & Hospital, Era University, Lucknow, India; ^5^ Division of Cardiovascular Medicine, Department of Medicine, University of Utah, Salt Lake City, UT, United States

**Keywords:** regional wall motion abnormality, RWMA, hypokinesia, anterior STEMI, echocardiography, electrolytes

## Abstract

Assessing regional wall motion abnormalities (RWMA) in the myocardium may provide early diagnosis and treat chronic remodeling in STEMI patients. We assessed RWMA in 217 subjects with anterior STEMI admitted to Era University Hospital in Lucknow, UP, India. Besides abnormalities in the LAD territory, sub-sets of patients exhibited diffuse regional myocardial dysfunction. Interestingly, variations in serum electrolytes, specifically sodium and potassium, significantly affected the distribution and frequency of RWMA. Notably, RWMA occurred in the basal septum, apical septum, apex, and lateral wall in the anterior STEMI group. Additionally, the rate of regional dysfunction varied with serum urea and creatinine levels. This suggests that anterior STEMI can manifest myocardial abnormalities beyond the LAD territory. These findings indicate that ST-segment elevation might not be specific, possibly influenced by electrolyte changes affecting cardiac rhythm. Therefore, diagnosing and correcting region-specific wall motion abnormalities and electrolyte imbalances may improve outcomes in STEMI patients.

## 1. Introduction

Regional wall motion abnormality (RWMA) predicts long-term mortality in coronary heart patients (1–3). Diagnosing heart failure (HF) with preserved ejection fraction (HFpEF) is challenging due to the lack of standardized criteria for myocardial dysfunction (4, 5). ST-elevation myocardial infarction (STEMI), the most severe acute coronary syndrome (ACS), rapidly reduces blood flow to the heart, mainly affecting the lower chamber (6, 7). However, STEMI patients fail to display the same physiology (8, 9). ECG sensitivity in detecting severe myocardial structural abnormalities in STEMI is low (10). In anterior STEMI, the left anterior descending (LAD) artery is most affected, while inferior or lateral STEMI involves the right coronary artery (RCA) and left circumflex (LCX) artery (11, 12). ST-segment elevation typically indicates total artery blockage (6, 13), which is insufficient to assess the overall cardiac pathology. Thus, collecting information about remodeling in various regions is vital for making informed decisions during emergency care.

In addition, it is essential to note that the cardiac action potential differs from the surface electrocardiogram, which represents the heart's total electrical activity recorded from the body surface (14). Action potentials also vary within the heart due to the presence of different ion channels in cardiac muscle (15). Furthermore, the action potential of cardiomyocytes in the myocardium varies based on an individual's cardiac health status (16), and the cardiac rhythm is influenced by electrolytes, including sodium (Na+), potassium (K+), and calcium (Ca2+) (17).

Given the significant variation in the cell-types in the myocardium across different heart regions (18, 19), it is crucial to explore the impact of electrolytes and the potential interference of other serum factors that might affect the action potential in myocardial flow. This study aims to characterize the prognostic significance of RWMA concerning changes in serum electrolytes in STEMI patients of North Indian origin.

## 2. Methodology

### 2.1. Human subjects

In the present study, we have assessed the incidence of RWMA in 217 anterior STEMI subjects admitted to the Era's Lucknow Medical College & Hospital, Era University by ECG, 2D-ECHO, and correlated with the serum sodium, potassium, urea, and creatinine (Refer to Supplementary Table S1).

### 2.2. Risk stratification in STEMI subjects

ST-elevation (STE) is the primary immediately available marker for detecting complete coronary artery occlusion without collateral circulation, indicating a significant region of injured myocardium at risk of irreversible infarction, necessitating urgent reperfusion therapy. Patients with acute chest pain were assessed based on ECG criteria for ST-elevation myocardial infarction (STEMI), as defined by the American College of Cardiology, American Heart Association, European Society of Cardiology, and the World Heart Federation committee. STE is considered significant when the J point of at least 2 contiguous leads measures ≥2 mm (0.2 mV) in men or ≥1.5 mm (0.15 mV) in women, in leads V2–V3 and/or ≥1 mm (0.1 mV) in other contiguous chest or limb leads (20). Reciprocal changes (ST depression in a region opposite the major vessel of injury) enhance STE specificity in STEMI. A new left bundle branch block is regarded as a STEMI equivalent. In leads V2-V3, the cutoff point is >0.2 mV in men over 40 years, and >0.25 in men under 40 years; >0.15 mV in women is considered STEMI. ST-segment elevation of 1.0 mm or more aligns with the QRS complex. Pre-existing left bundle branch block cases were further assessed using Sgarbossa's criteria (21).

#### 2.2.1. Inclusion criteria for the study

All subjects were between the ages of 30 and 95. Subjects with anterior STEMI, i.e., the occurrence of ≥1 and stenosis of ≥50% in ≥1 of 15 coronary segments were selected for the study.

#### 2.2.2. Exclusion criteria for the study

Individuals with stenosis <50%, septicemia, acute and chronic kidney disease, cerebrovascular accident, presence of malignancy, and pregnant women were excluded from the study.

### 2.3. Electrocardiography

ECG recordings and analysis were performed following the American College of Cardiology, American Heart Association, European Society of Cardiology, and the World Heart Federation committee criteria using GE MAC, 2000 12 lead machine. The rate of ECG acquisition was set at 25 mm/sec with the voltage set at 10 mm/millivolt. The standard 12 lead ECG was a 10-second strip. The bottom line was a full rhythm strip spanning the whole 10 s of the ECG - other leads spanned only about 2.5 s.

### 2.4. Two-dimensional echocardiography

Two-dimensional echo and doppler analyses were performed using PHILIPS Epic 7C system. ECHO images were acquired using parasternal long axis view, parasternal short axis view, apical four-chamber view, apical two-chamber view, and apical five-chamber view. Regional wall motion was recorded using 16 – Segment model, recommended by the American Society of Cardiology. Ejection fraction (EF) and segmental wall motion tracing were analyzed in PSLAX M-Mode of LV dimensions from mid-ventricular papillary muscle level. Stroke volume (SV) is calculated as the difference between end-diastolic volume (EDV) and end-systolic volume (ESV). LVEF is calculated using the formula LVEF: (SV/EDV)×100. For assessing longitudinal contraction, Simmons method was adopted to trace the LV endocardial border in both the apical four-chamber and two-chamber views in end-systole and end-diastole.

### 2.5. Biochemical parameters

Serum - Sodium (Na+) and Potassium (K+) concentrations were quantitatively measured using commercial kits (VITROS). Na+ Slides and K+ Slides (Potentiometric Micro Slide Kit); Serum-urea and – creatinine were quantitatively measured by VITROS - urea slides and creatinine slides (Colorimetric Micro Slide Kit) as described by the manufacturer.

## 3. Results

### 3.1. Impact of RWMA on ejection fraction in STEMI patients

In this cross-sectional study, the age distribution of both female and male STEMI patients is comparable (54.4 ± 9.3 vs. 53.9 ± 10.7, *p* > 0.05, ns) (Supplementary Table S1). The study involved 217 anterior STEMI patients aged 30–91 years. Approximately 60% of males and 70% of females fell within the 51–91 age group, while 40% of males and 30% of females were in the 30–50 age group. The baseline characteristics of the patients are presented in Supplementary Table S1.

**Figure 1:**
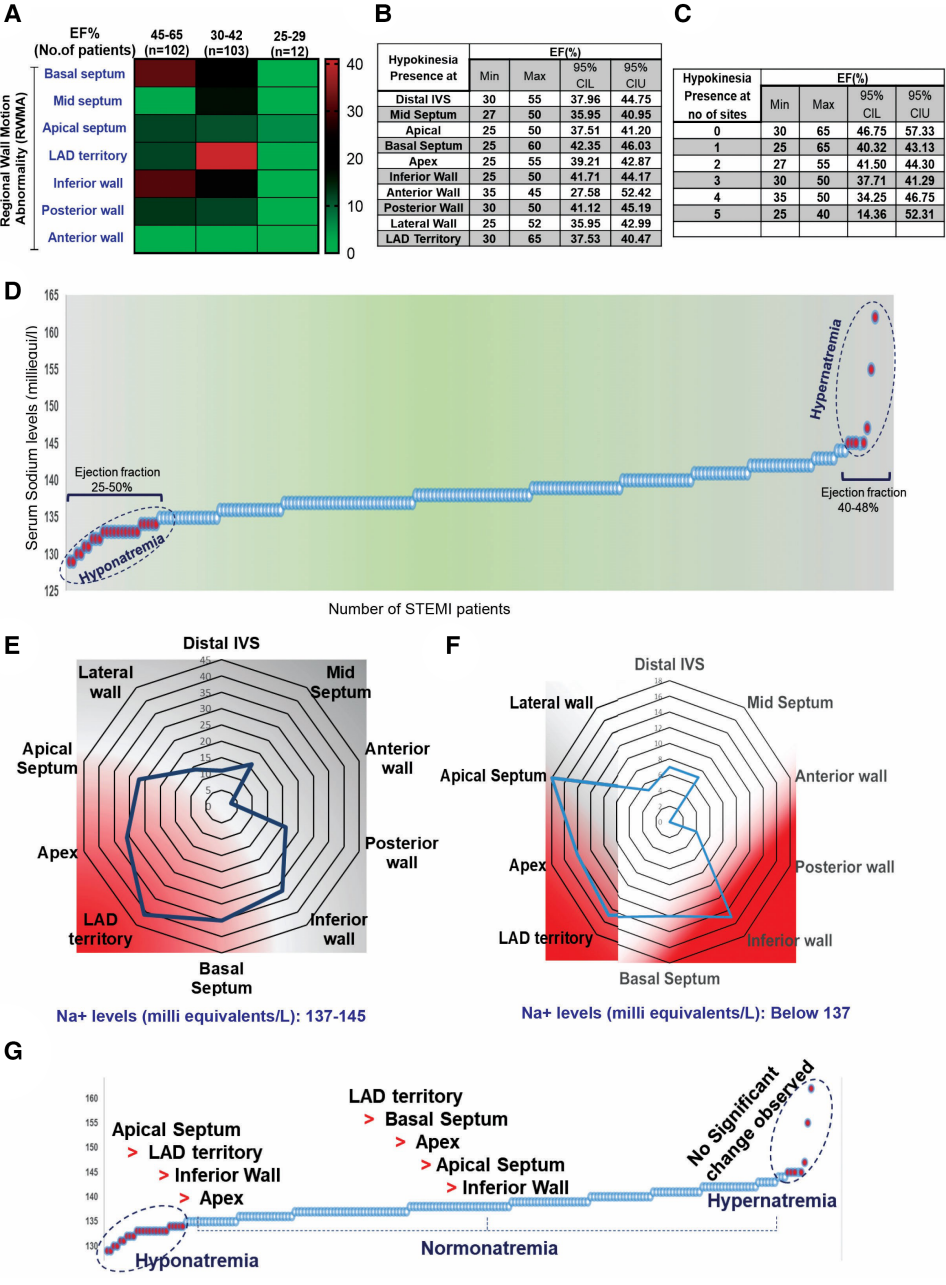
Baseline characteristics of the patients involved in the present study. (**A**) Distribution of RWMA in STEMI patients (*n* = 217) with normal EF. (**B,C**) 95% CI comparison between EF and incidence of RWMA sites in STEMI. (**D**) Multivariate representation of RWMA with EF and serum sodium levels in STEMI patients. Serum sodium levels and occurrence of STEMI; (**E,F**) Incidence of RWMA in different myocardial zones (LAD, basal septum, apex, apical septum, inferior wall) with respective of hypo and hyper-natremia. (**G**) Prevalence of hypokinetic segments scored based on the circulatory levels of sodium.

Among the 217 STEMI subjects, 92% exhibited regional wall motion abnormalities (RWMA) at multiple sites, including the basal septum (25%), mid septum (11%), apical septum (20%), LAD territory (28%), inferior wall (23%), posterior wall (12%), and anterior wall (1.8%), regardless of the ejection fraction (EF). Variable degrees of RWMA with multiple overlapping zones were identified in STEMI patients with normal EF (Figure 1A). Analysis of specific regional walls revealed significant changes in EF, with a minimum EF of 35% (95% CI: 27.58%–52.42%) observed in the anterior wall and a maximum EF of 65% (95% CI: 37.53%–40.47%) found in the LAD territory. In 182 STEMI subjects, regional wall abnormality was observed in less than two sites, and the minimum EF was 25%. In three subjects, five sites were affected, and the EF was less than 40% (Figures 1B,C).

### 3.2. Interdependence of serum sodium levels and RWMA

The serum sodium level is an independent clinical variable for identifying HF with preserved ejection fraction (HFpEF) (22–24). In our study, we did not find an interdependence of serum sodium, ejection fraction, and the incidence of STEMI complications (Supplementary Table S2). However, we observed that the degree of RWMA varied in STEMI subjects in accordance with hypo-, normal-, and hyper-natremia grades (Figures 1D–F). We scored the hypokinetic segments based on serum sodium levels and found that regional dysfunction was more prominent in the hyponatremic group, with the order being apical septum > LAD > inferior wall > apex, compared to the normal-natremia group, which followed the order LAD > basal septum > apex > apical septum > inferior wall. There were no significant RWMA observed in the hypernatremia group (Figure 1G). These observations strongly support the influence of sodium levels on RWMA.

### 3.3. Interdependence of serum potassium levels and RWMA

**Figure 2:**
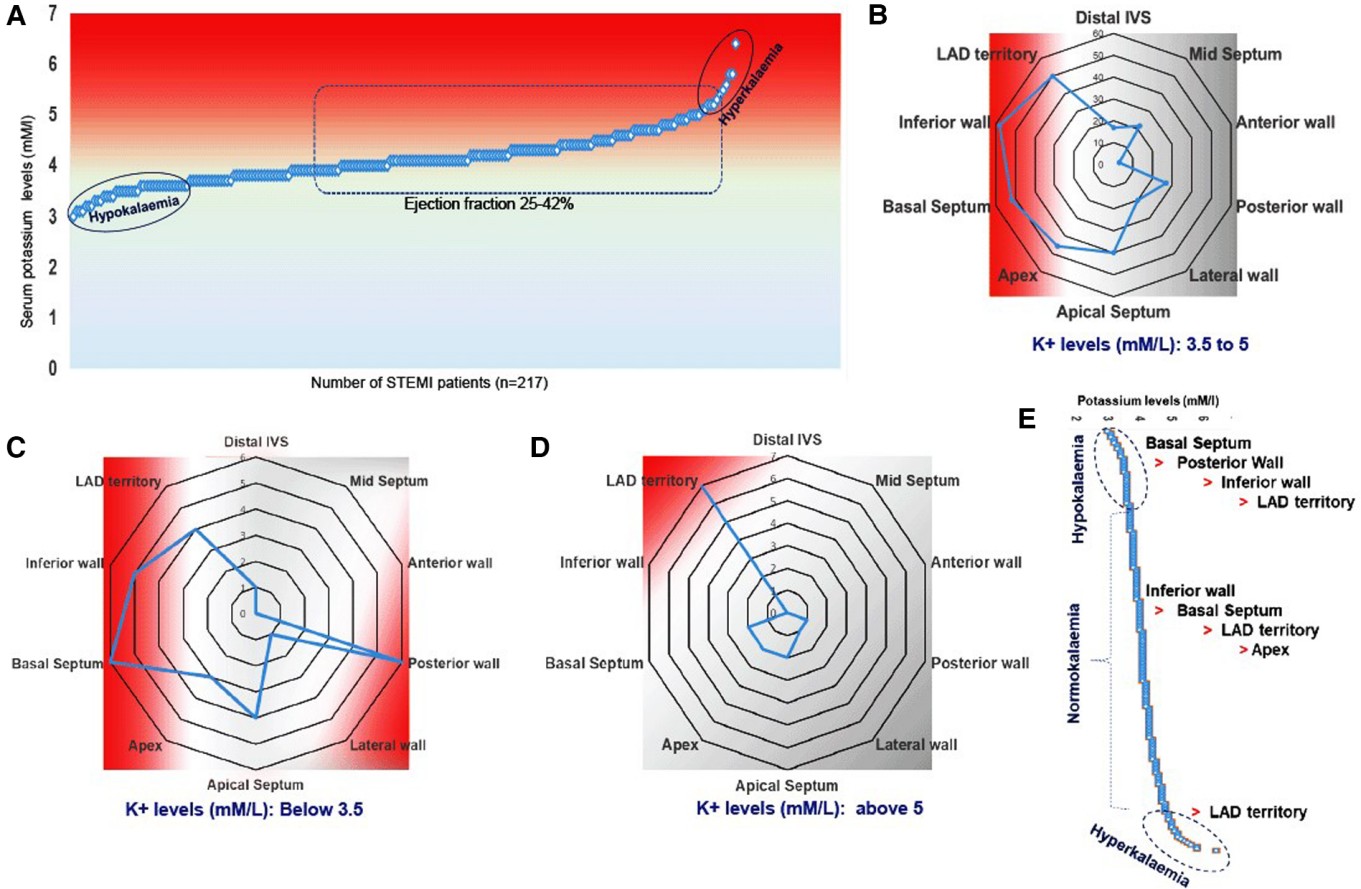
Multivariate representation of RWMA with EF and serum potassium levels in STEMI patients. (**A**) Serum potassium levels and occurrence of STEMI; (**B–D**) Incidence of RWMA in different myocardial zones (LAD, basal septum, apex, apical septum, inferior wall) with respective of hypo and hyper-kalemia. (**D**) Prevalence of hypokinetic segments scored based on the circulatory levels of potassium.

Both reduced (hypokalemia) and increased (hyperkalemia) serum potassium levels are associated with cardiac arrhythmia (25, 26). In our study, we found no association between baseline serum potassium levels, EF, or STEMI (Figure 2A). Neither the lowest nor the highest serum sodium levels could predict the occurrence of STEMI complications (Figure 2A). However, we observed that hypokalemia was more prevalent than hyperkalemia. The shift in baseline serum potassium concentration toward hypokalemia was linked to RWMA, with the order being basal septum > posterior wall > inferior wall > LAD territory (Figure 2B), in comparison with the normokalemia group, where the order was inferior wall > basal septum > LAD territory > apex (Figure 2C). In STEMI subjects with hyperkalemia, LAD territory abnormality was more common (Figures 2D,E).

### 3.4. Differential inference of serum urea levels on RWMA

**Figure 3:**
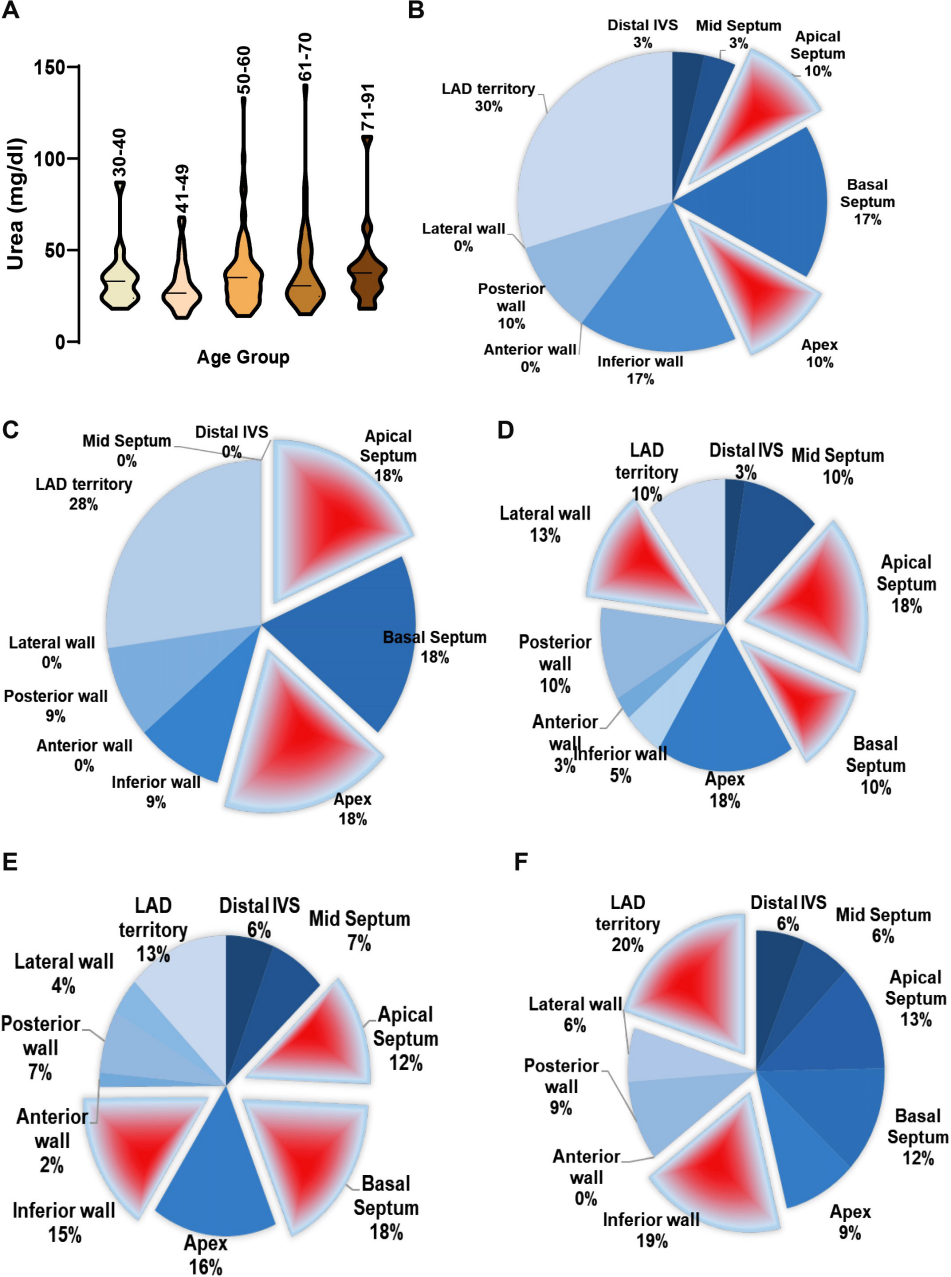
Multivariate representation of RWMA with serum urea levels in STEMI patients. (**A**) Serum urea concentration in STEMI patients categorized under different age groups; Incidence of RWMA in different myocardial zones (LAD, basal septum, apex, apical septum, inferior wall) with respective to serum urea levels in STEMI in females (**B,C**) and males (**D–F**).

We found a poor correlation between serum urea and EF (Supplementary Tables S3,S4 and Figure 3A). Nonetheless, with increasing age, urea levels in STEMI patients appear to rise, possibly indicating pre-onset kidney dysfunction. In female STEMI patients with hyperurea, we observed prominent hypokinesia at the basal septum (17%), inferior wall (17%), and LAD territory (30%) (Figure 3B). Interestingly, another group of STEMI subjects with normal urea levels showed a high prevalence of RWMA at the apical septum (20.3%), apex (26.3%), basal septum (25.3%), and LAD territory (28%), while the inferior wall exhibited less prominence (9%) (Figure 3C). This suggests the potential influence of other serum electrolytes on myocardial action potential.

Among males with low urea levels, significant RWMA was found at the apical septum (18%), lateral wall (13%), and basal septum (10%), with lower frequency in the LAD territory (Figure 3D). In contrast, male STEMI patients with normal urea levels exhibited RWMA primarily in the basal septum (18%), inferior wall (15%), apical septum (12%), and apex (16%) (Figure 3E). In males with hyperurea, the area of abnormality shifted to the LAD territory (20%) and inferior wall (19%), involving other areas as well (Figure 3F).

### 3.5. Impact of creatinine levels on RWMA display gender disparities

**Figure 4:**
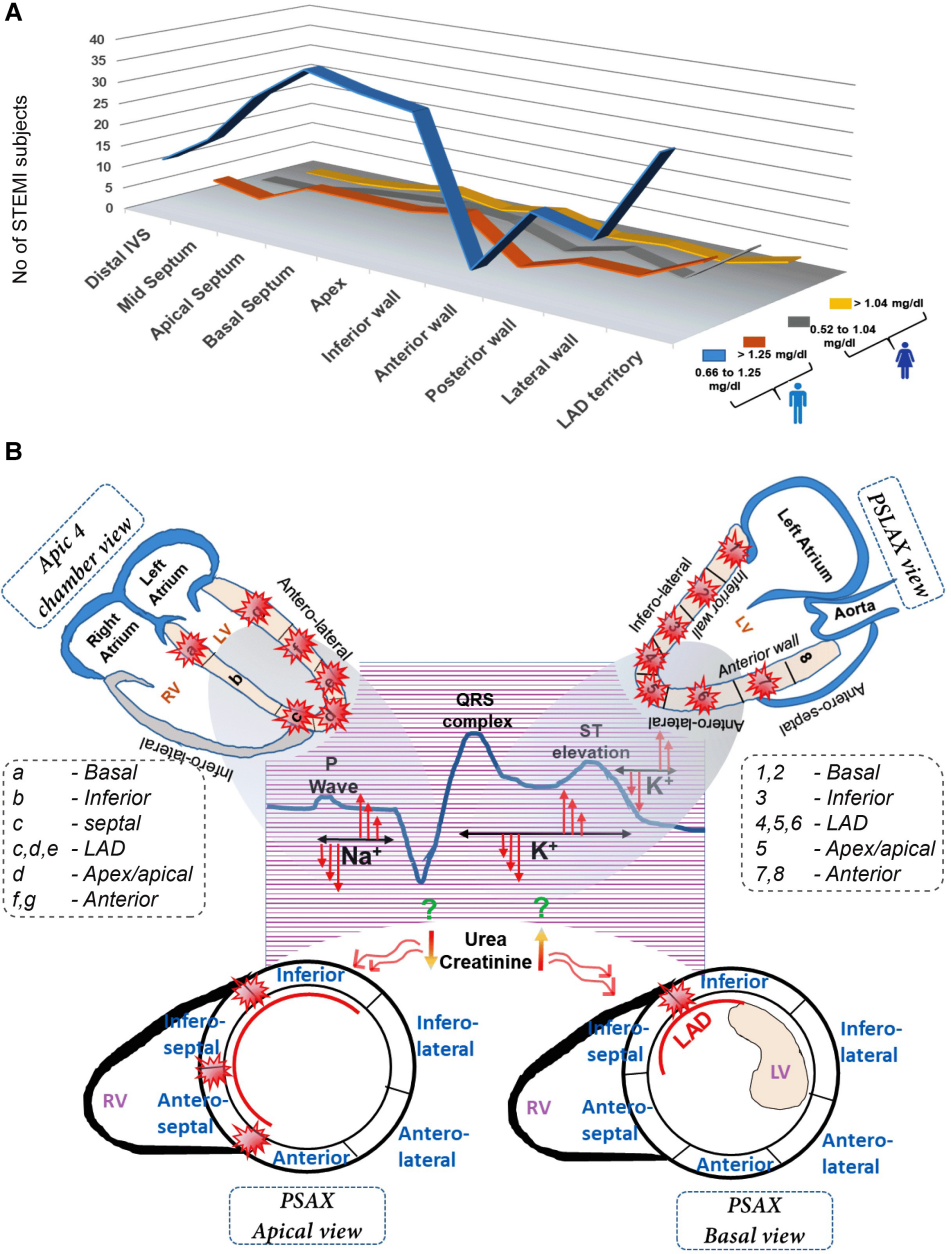
Multivariate representation of RWMA with serum creatinine levels in female (**A**) and male STEMI patients (**B**) A comprehensive view of differential impact of serum electrolyte changes in the onset of STEMI pattern and its subsequent influence on wall motion abnormalities in distinct myocardial segments.

Interestingly, males with normal creatinine levels exhibit prominent RWMA at the apical septum, apex, lateral wall, and LAD territory, while males with high creatinine levels display predominant but insignificant abnormalities in the LAD territory. Females with both high and normal serum creatinine levels do not show any significant or prominent areas with RWMA (Figure 4A). No direct correlation exists between EF and creatinine levels (Supplementary Tables S3,S4).

## 4. Discussion

ECG patterns/scores have been used to classify STEMI subjects (27). However, echocardiography can detect regional or segmental wall motion abnormalities more sensitively and serve as a specific tool to predict STEMI onset (28). In this cross-sectional study, despite EF, gender, and age, all 217 STEMI subjects displayed diffuse regional myocardial dysfunction. Among them, 103 exhibited severe abnormality in the left anterior descending coronary artery territory (LAD), and 102 showed hypokinesia at the basal septum and inferior wall. This higher incidence of hypokinesia in the LAD territory slightly correlated with STEMI patients having EF between 30% and 40%. It is thought that increased LAD wall motion abnormality might result from reduced collateral blood supply, leading to scar formation (29, 30).

Electrolyte imbalances are common in STEMI subjects (31, 32), and heart rhythm is influenced by electrolyte concentration (33). Although hyperkalemic STEMI is rare (34), our study revealed that 28.1% of STEMI subjects with hyperkalemia had RWMA in the LAD territory. This observation of hyperkalemia along with severe hypokinesia in the LAD territory is novel in STEMI subjects. High potassium levels may shorten the action potential during phase three repolarization, potentially leading to STEMI (35, 36).

Serum urea and creatinine levels have the potential to predict kidney dysfunction in coronary patients (37). However, the direct correlation between serum urea/creatinine and the incidence of coronary complications is not well understood. To date, there have not been parallel studies investigating the possible impact of urea and creatinine on myocardial regional wall abnormalities.

In this study, we observed variations in serum electrolytes, such as sodium and potassium, resulting in differences in the distribution and frequency of regional abnormalities among STEMI subjects (Figure 4B). This suggests that the anterior STEMI category should not be limited to abnormalities in the LAD territory alone; other myocardial regions must also be considered for classification. We propose that ST-segment elevation might not be specific and could be caused by non-cardiac factors like electrolyte imbalances. Therefore, a specific and predictable diagnostic approach is necessary for identifying the STEMI patients. While a comprehensive analysis may delay immediate therapy, it is crucial to detect RWMA using echocardiography for personalized care post-MI. Understanding common electrolyte abnormalities in conjunction with RWMA incidence can aid in interpreting STEMI presentations and expediting emergency cardiac care, leading to more appropriate diagnostic and therapeutic measures. Additionally, classifying STEMI subjects based on the site of RWMA and correcting electrolyte imbalances may lead to better outcomes for survivors.

### 4.1. Clinical implications

This study emphasizes improved diagnostic methods for STEMI patients, using sensitive echocardiography to assess myocardial health. Widespread myocardial dysfunction is observed, particularly in the LAD territory. Monitoring and correcting electrolyte imbalances are crucial. Combining electrolyte correction with traditional PCI interventions expedites patient recovery. Personalized care based on RMWA and electrolyte correction may significantly improve outcomes.

### 4.2. Limitation

This study offers valuable insights but comes with several limitations. Firstly, being a cross-sectional study, unable to establish causal relationships. To investigate these associations, we need longitudinal studies with a larger patient cohort. Secondly, it focuses on a specific ethnic population, and require more diverse and extensive samples for generalizing. Furthermore, the specific mechanisms behind the associations between electrolytes, myocardial abnormalities, and STEMI remain unclear in this study.

## Data Availability

The original contributions presented in the study are included in the article/**Supplementary Material**, further inquiries can be directed to the corresponding author at the study site, Dr. Farzana Mahdi & Dr. S. Mohd. Shiraz Rizvi.
