# Supplementary material for: Influence of electrolyte imbalance on regional wall motion abnormalities in STEMI patients of North Indian origin
Source: Front Cardiovasc Med. 2023 Nov 30;10:1223954. doi: 10.3389/fcvm.2023.1223954 (PMC10720728; doi:10.3389/fcvm.2023.1223954)
Supplement: Supplementary file 1 [file Datasheet1.pdf]

Supplemental Section

Prevalence of distinct regional wall motion abnormalities among STEMI patients of north Indian origin

S. Mohd. Shiraz Rizvi[#], Sini Sunny[#], Irshad A. Wani, Farzana Mahdi, Zeeshan H. Zaidi, Namakkal S. Rajasekaran

Suppl Table I

Patient Demographics

Variable	Mean ± SD
Age (Years)	54±10.5
Sex (M/F)	186/31
HB (g/dl)	12.4±1.7
TLC (cells/cumm)	8934±2501.8
PC (x10^6 cells)	2.1±0.7
MCV (fl)	84.5±8.0
Na+ (mmol/L)	137.6±10.0
K+ (mmol/L)	4.4±4.5
Urea (mg/dl)	35.9±19.0
Creatinine (mg/dl)	1.0±0.5
SGOT (U/L)	114.8±149.4
SGPT (U/L)	60.6±76.3
S ALK (U/L)	100.9±33.4
S PRO (U/L)	6.7±0.0

Suppl Table II

Interdependence of serum parameters with EF

Parameter	EF <30%		EF : 30% - 42%		EF : > 45% - 65%		ANOVA	
	Mean	SD	Mean	SD	Mean	SD	F-value	p-value
HB	12.69	1.34	12.33	1.82	12.50	1.69	0.41	0.662
TLC	8715.38	2726.98	9181.00	2611.50	8719.61	2362.33	0.91	0.404
PC	2.08	0.83	2.06	0.74	2.09	0.68	0.04	0.959
MCV	80.12	14.42	85.20	7.20	84.42	7.54	2.38	0.095
Na+	136.46	3.69	136.46	14.10	138.93	3.26	1.66	0.192
K+	4.34	0.63	4.69	6.52	4.15	0.51	0.37	0.693
UREA	50.77	31.67	38.42	19.12	31.41	15.29	8.21	**0.000**
CREATININE	1.12	0.30	1.11	0.58	0.96	0.29	3.07	**0.049**
SGOT	117.33	171.64	140.32	178.66	87.01	100.45	2.97	0.054
SGPT	128.00	258.70	59.94	51.95	52.00	28.91	5.49	**0.005**
S ALK	94.42	23.38	102.95	36.49	99.54	31.10	0.47	0.623

Suppl Table III

Multinomial Logistic Regression Analysis to Predict EF% on the basis of Likely Significant Predictors

Reference: EF: = 45%	Predictor Variable	B	SE	p-value	Exp(B)	95% CIL for Exp(B)	95% CIU for Exp(B)
EF <30%	Intercept	10.81	9.11	0.235			
	MCV	-0.05	0.03	0.092	0.95	0.89	1.01
	Na	-0.07	0.06	0.228	0.93	0.83	1.05
	UREA	0.04	0.02	**0.036**	1.05	1.00	1.09
	CREATININE	-0.39	1.49	0.797	0.68	0.04	12.72
	SGOT	0.00	0.00	0.932	1.00	1.00	1.01
	SGPT	0.00	0.00	0.321	1.00	1.00	1.01
EF: 30% - 42%	Intercept	10.65	6.33	0.093			
	MCV	0.01	0.02	0.608	1.01	0.97	1.05
	Na	-0.09	0.04	**0.040**	0.92	0.84	1.00
	UREA	0.01	0.01	0.400	1.01	0.99	1.04
	CREATININE	0.15	0.74	0.836	1.17	0.27	4.98
	SGOT	0.00	0.00	**0.034**	1.00	1.00	1.01
	SGPT	0.00	0.00	0.693	1.00	0.99	1.01

Suppl Table IV

RWMA	No RWMA		RWMA		unpaired t test	
	Mean	SD	Mean	SD	t-value	p-value
HB	12.70	1.88	12.41	1.72	0.62	0.535
TLC	9253.33	1816.14	8910.00	2547.68	0.51	0.609
PC	1.99	0.50	2.08	0.73	-0.46	0.643
MCV	85.92	5.68	84.42	8.14	0.70	0.485
Na+	140.20	2.91	137.43	10.30	2.65	**0.011**
K+	4.23	0.65	4.43	4.63	-0.16	0.872
UREA	30.33	15.73	36.26	19.22	-1.39	0.184
CREATININE	0.93	0.28	1.05	0.47	-0.93	0.352
SGOT	74.60	91.81	118.20	152.96	-1.09	0.279
SGPT	53.00	36.82	61.18	78.54	-0.39	0.700
S ALK	92.86	22.36	101.52	34.08	-0.93	0.352

Suppl Table V

Binary Logistic Regression Analysis to Predict RWMA on the basis of Likely Significant Predictors

Predictors	B	SE	p-value	OR	95% CIL	95% CIU
Na	-0.13	0.07	0.059	0.88	0.77	1.00
UREA	0.02	0.02	0.410	1.02	0.98	1.06
SGOT	0.00	0.00	0.356	1.00	1.00	1.01
Constant	19.38	9.50	0.041	Large	-	-
